# Effects of Complex Modification by Sr–Sb on the Microstructures and Mechanical Properties of Al–18 wt % Mg_2_Si–4.5Cu Alloys

**DOI:** 10.3390/ma9030157

**Published:** 2016-03-04

**Authors:** Youhong Sun, Shaoming Ma, Huiyuan Wang, Lei Chen, Ke Gao, Yinlong Ma, Baochang Liu

**Affiliations:** 1 School of Construction Engineering, Jilin University, Changchun 130026, China; syh@jlu.edu.cn (Y.S.); masm14@mails.jlu.edu.cn (S.M.); ylma@jlu.edu.cn (Y.M.); 2 Key Laboratory of Drilling and Exploitation Technology in Complex Conditions, Ministry of Land and Resources, No. 938 Ximinzhu Street, Changchun 130026, China; 3 Key Laboratory of Automobile Materials of Ministry of Education & School of Materials Science and Engineering, Jilin University, No. 5988 Renmin Street, Changchun 130025, China; wanghuiyuan@jlu.edu.cn (H.W.); chenl11@mails.jlu.edu.cn (L.C.)

**Keywords:** aluminum alloy, composite, grain refinement, modification, mechanical properties

## Abstract

This research was carried out to investigate the influence of Sr–Sb on the microstructures and mechanical properties of Al–18 wt % Mg_2_Si–4.5Cu alloys. After the addition of 0.2 wt % Sr–Sb, the morphologies of primary Mg_2_Si transformed from equiaxed dendrite to cube in as-cast alloys and the average size of primary Mg_2_Si decreased from ~50 to ~20 μm. The shape of eutectic Mg_2_Si changed from Chinese script to short rod. After extrusion and T6 heat treatment, the ultimate tensile strength of modified alloy at room temperature (RT) and 100 °C increased respectively from 229 to 288 MPa, and from 231 to 272 MPa. The elongation-to-failure only slightly improved from 2.9% to 3.8% and from 3.3% to 3.7% at RT and 100 °C, respectively. The tensile fracture surface revealed a transition from brittle fracture to ductile fracture after modifying by 0.2 wt % Sr–Sb.

## 1. Introduction

The development of high strength aluminum alloys becomes significant to promote the application of aluminum alloys in transport and other fields where improved strength and light weight are essential. Recently, metal matrix composites (MMCs) have been rapidly replacing conventional materials in various engineering applications [1]. The intrinsic advantage of MMCs over the unreinforced alloy is the improvement of high temperature mechanical properties and wear resistance.

Mg_2_Si is an intermetallic compound which is frequently used to reinforce the light alloys. Mg_2_Si exhibits a high melting temperature (1085 °C), a low density (1.99 × 10^3^ kg·m^−3^), a high hardness (4.5 × 10^3^ MPa), a low coefficient of thermal expansion (7.5 × 10^−6^ K^−1^) and a high elastic modulus (120 GPa) [2]. Furthermore, the Mg_2_Si phase is exceptionally stable and could effectively impede grain boundary sliding at elevated temperatures [3]. It was proved that Mg_2_Si reinforced Al composites have improved mechanical properties at a wide range of temperatures [3,4] and high wear resistance [5]. It is also attractive in materials because of the abundance and nontoxicity of its constituent elements [6]. Owing to the excellent combination of physical and mechanical properties, Mg_2_Si has been widely used as a reinforced phase to prepare Mg_2_Si/Al alloys, which are attractive candidate material for aerospace, automotive, and other applications such as drilling pipes in deep and ultra-deep wells for gas and oil [7,8,9,10].

Unfortunately, the as-cast Mg_2_Si reinforced alloys produced by conventional gravity casting tends to form undesirable, coarse dendritic primary morphology and brittle Chinese script eutectic structures, which would damage the mechanical properties of the alloys and severely limit their development and applications [9]. During the past two decades, many attempts have been made to explore the relationship between the microstructure and deformation behavior in particulate reinforced metal matrix composites. It has been shown that the mechanical behavior of this kind of composite material is significantly affected by their microstructure such as Young’s modulus of the particles, particle aspect ratio, particle size and volume fraction, as well as the strain-hardening exponent of the matrix material [11,12]. As a consequence, controlling the morphology and the size of Mg_2_Si are the main objects in Mg_2_Si reinforced light alloys.

Modification is an effective method to control the morphology and size of Mg_2_Si, through which the growth of Mg_2_Si can be restricted by poisoning the surface of Mg_2_Si with the help of certain modifying elements. Up to now, the modification and refinement mechanisms of the Mg_2_Si by adding various elements in Al–Mg–Si alloys and Mg–Al–Si alloys have been discussed in the previous studies. Yang *et al.* [13] reported that after adding 0.09 wt % Sr to AZ61–0.7Si alloys, the morphology of eutectic Mg_2_Si changed from coarse Chinese script shape to fine granule and/or irregular polygonal shape, which was attributed to both restricting growth effect and increasing undercooling degree. Alizadeh *et al.* [3] found that the morphology of Chinese script eutectic Mg_2_Si phase in as-cast Mg–4Zn–2Si alloys could be changed to rounded edge shape with 0.2 wt % Sb addition and significantly improve its creep resistance, shear strength and hardness at elevated temperature. Li *et al.* [14] suggested that 3 wt % P addition can obtain favorable refining and modifying effect in as-cast Al–*x*% Mg_2_Si (*x* = 15, 20, 30) alloys because Mg_2_Si may nucleate on AlP surface. Qin *et al.* [15] found that the primary Mg_2_Si changed from dendrite to polygon in the Al-25Mg_2_Si-3Si-3Cu composite when modified by 0.5 wt % P and aged, accompanied by an increase in ultimate tensile strength (UTS) from 190 to 249 MPa. Recently, N. Soltani *et al.* [4] investigated the effect of Ti concentrations on the microstructure and tensile properties of an in-situ Al-Mg_2_Si composite; the highest UTS and elongation values were found to be 245 MPa and 9.5% for homogenized and extruded Al-15Mg_2_Si-0.5Ti composite at room temperature. Therefore, it is of great importance and interest to investigate the influence of modifiers on the mechanical properties of Al-Mg_2_Si alloys for better understanding the microstructure-mechanical properties relationship.

Unfortunately, most of the present results are mainly restricted to single modifying elements. Little study on combined addition (two or more elements) are reported [16]. Wang *et al.* [9] reported for the first time that after combined additions of Sr and Sb, most primary Mg_2_Si crystals in Mg–*x* (*x* = 3, 5) Si–Al alloys, transformed from equiaxed-dendritic shapes to octahedral morphologies, while eutectic phases also changed from Chinese script to short rod-shapes. The mechanisms of complex modification of Sr–Sb were attributed to the heterogeneous nucleation of primary Mg_2_Si on Sr_11_Sb_10_ nucleus, together with change in growth manners caused by incorporation of Sb in Mg_2_Si crystals. However, there is no report on the effect of complex modification by Sr–Sb on the morphology of Mg_2_Si in Al–Cu alloys to date. As a result, the present study is aimed to investigate the effect of combined addition of Sr–Sb on the Mg_2_Si morphology in Al–18Mg_2_Si–4.5Cu alloys. The mechanical properties of the hot-extruded and T6 heat treatment alloys are examined, and the strengthening mechanisms are also discussed. It is expected that the preliminary results could be helpful in controlling the morphology and size of Mg_2_Si reinforcement and promoting the development of high quality Mg_2_Si reinforced Al–Cu alloys.

## 2. Materials and Methods

Al–24.4Si alloys ingot, pure Al ingot (99.98 wt % purity), Mg ingot (99.85 wt % purity) and Cu rod (99.90 wt % purity) were used as starting materials to prepare Al–18Mg_2_Si–4.5Cu alloys. Al–10Sr master alloy rod and pure Sb nuggets (98.00 wt % purity) were used as modifiers. First, Al–24.4Si alloys and pure Al were melted at 720 °C in a clay crucible in an electric resistance furnace of 5 kW. Then pure Mg and Cu preheated at 200 °C in the box-type resistance furnace were added to the melt. The melts were manually stirred for about 2 min to facilitate the incorporation and uniform distribution of Mg and Cu in melts. After that, the melts were held at 720 °C for about 20 min, during which time the melts were stirred every 5 min and then deslagged before finally being poured at 720 °C into a copper mold which had been preheated at 200 °C to produce cast ingot that was 30 mm in diameter and 140 mm in height. The Al–18Mg_2_Si–4.5Cu–0.2 (Sr–Sb) alloys were prepared in the same way except adding Al–10Sr and pure Sb which were preheated at 200 °C. The designed composition of Sr–Sb in melt was 0.2 wt %, with an atom ratio of 11:10. The addition of 0.2 wt % Sr–Sb, which can effectively modify the primary and eutectic Mg_2_Si, is chosen based on the Ref. [9]. Phase constituents of samples were analyzed by X-ray diffraction (XRD) (D/Max 2500PC, Rigaku Ltd., Tokyo, Japan) using Cu Kα radiation in step mode from 20° to 80° with a scanning speed of 4°/min. Metallographic samples with the sizes of 10 mm × 10 mm × 10 mm were prepared in accordance with standard procedures used for metallographic preparation of metal samples and etched with 0.5% HF (in vol.) for about 15 s at room temperature. The primary and eutectic Mg_2_Si were extracted with 10% (in vol.) HNO_3_ solutions from the Al–18Mg_2_Si–4.5Cu and Al–18Mg_2_Si–4.5Cu–0.2(Sr–Sb) alloys. Microstructure and phase analyses were investigated by optical microscopy (OM) (Carl Zeiss–Axio Imager A2m, Carl Zeiss AG, Gottingen, Germany), and scanning electron microscopy (SEM) (ZEISS EVO18, Carl Zeiss AG, Mainz, Germany). Moreover, the morphologies of the extracted primary and eutectic Mg_2_Si were observed using a field emission scanning electron microscope (FESEM) (JSM–6700F, JEOL, Tokyo, Japan).

Cylinder samples were cut manually with a size of 30 mm in diameter and 30 mm along longitudinal axis for the extrusion process. The samples were homogenized at 420 °C for 4 h and followed by furnace cooling. Then the extrusion experiment was carried out at 440 °C with an extrusion ratio of 16:1 and followed by air cooling. After that the samples were solution treated at 500 °C for 2 h and then aged at 175 °C for 8 h. The tensile strength and fracture elongation were tested at room temperature and 100 °C by an electronic universal test machine (DDL 100, CIMACH, Changchun, China) at the speed of 0.18 mm/min, at least three tensile tests were done for each condition to ensure the accuracy of results. The fracture morphology was observed by SEM (EVO18, ZEISS, Carl Zeiss AG, Mainz, Germany). Moreover, the microhardness of as-cast and hot-extruded alloys were tested by Microhardness Tester (1600–5122VD Microment 5104, Buehler, Chicago, IL, USA) under an applied load of 50 g for 15 s on the Al matrix. At least seven measurements were done for each condition to ensure the accuracy of results.

## 3. Results and Discussion

**Figure 1:**
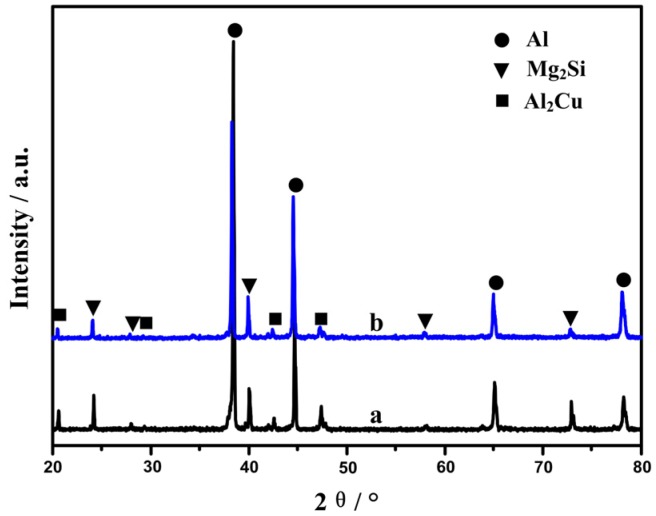
X-ray diffraction (XRD) results for as-cast alloys: (**a**) Al–18Mg_2_Si–4.5Cu; (**b**) Al–18Mg2Si–4.5Cu–0.2(Sr–Sb).

The Al, Mg_2_Si and Al_2_Cu phases in Al–18Mg_2_Si–4.5Cu alloys are identified by XRD analysis, as shown in Figure 1. The result reveals that the addition of 0.2 wt % Sr–Sb has no influence on phase compositions of the alloys, which is consistent with the previous study [17,18].

**Figure 2:**
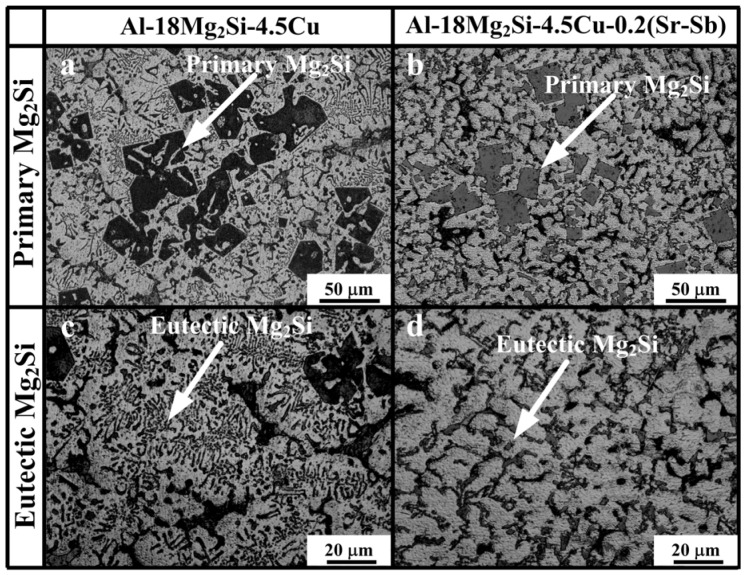
Microstructures of Mg_2_Si in as-cast alloys: (**a**) primary Mg_2_Si in Al–18Mg_2_Si–4.5Cu; (**b**) primary Mg_2_Si in Al–18Mg_2_Si–4.5Cu–0.2 (Sr–Sb); (**c**) eutectic Mg_2_Si in Al–18Mg_2_Si–4.5Cu; (**d**) eutectic Mg_2_Si in Al–18Mg_2_Si–4.5Cu–0.2 (Sr–Sb).

The combined addition of 0.2 wt % Sr–Sb causes an evident modification effect on both primary and eutectic Mg_2_Si in as-cast alloys. The OM microstructure of as-cast Al–18Mg_2_Si–4.5Cu alloys before and after adding 0.2 wt % Sr–Sb are shown in Figure 2a,d. It can be seen that the microstructure consists of coarse particles of primary Mg_2_Si (Figure 2a) and Chinese script eutectic Mg_2_Si (Figure 2c) in the Al–18Mg_2_Si–4.5Cu matrix. By adding 0.2 wt % Sr–Sb in Al–18Mg_2_Si–4.5Cu alloys, the morphology of primary Mg_2_Si transforms from equiaxed dendrite (Figure 2a) to polygon (Figure 2b), and the size of primary Mg_2_Si decreases from ~50 to ~20 μm. Eutectic Mg_2_Si changes from Chinese script (Figure 2c) to short rod shape (Figure 2d). It can be clearly seen that the modified eutectic Mg_2_Si has smaller sizes compared with the unmodified one. The modification mechanisms will be discussed later.

**Figure 3:**
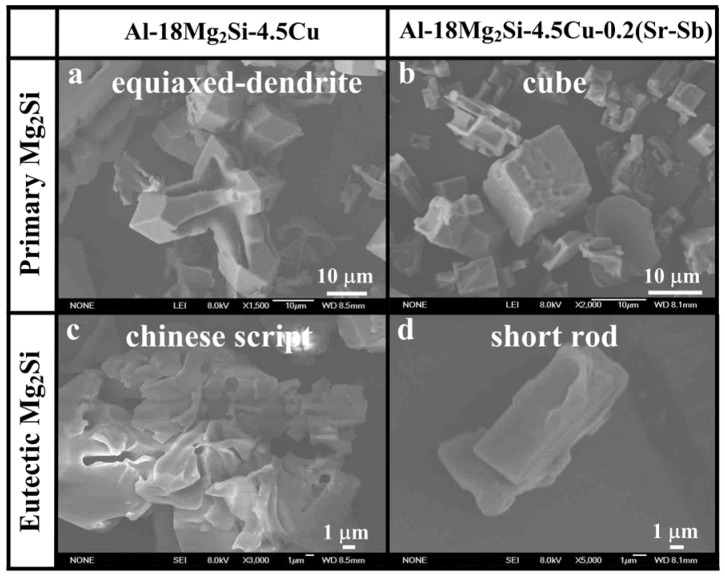
The field emission scanning electron microscope (FESEM) morphology of Mg_2_Si in as-cast alloys: (**a**) primary Mg_2_Si in Al–18Mg_2_Si–4.5Cu, (**b**) primary Mg_2_Si in Al–18Mg_2_Si–4.5Cu–0.2 (Sr–Sb); (**c**) eutectic Mg_2_Si in Al–18Mg_2_Si–4.5Cu; (**d**) eutectic Mg_2_Si in Al–18Mg_2_Si–4.5Cu–0.2 (Sr–Sb).

The representative FESEM images show that the three-dimensional morphologies of extracted primary Mg_2_Si changes from equiaxed dendrite (Figure 3a) to cube (Figure 3b) after the modification of 0.2 wt % Sr–Sb. Interestingly, the effect of the Sr–Sb complex modification on the morphology of primary Mg_2_Si are obviously different from that adding Sr and Sb in isolation.

According to the results of Wang *et al.* [9], we proposed that the modification mechanisms of Sr–Sb additions on primary Mg_2_Si crystals in Al–18Mg_2_Si–4.5Cu alloys are mainly related to the two aspects: during solidification, the Sr_11_Sb_10_ compounds were formed prior to primary Mg_2_Si crystals, which acted as a heterogeneous nucleus for the latter. On the other hand, some Sb atoms were incorporated in Mg_2_Si crystals by substituting Si sites, and thereby changing growth manners from initial equiaxed dendrite morphologies to cube. The modification and refinement mechanisms for combined addition of Sr–Sb are significantly different from those of adding Sr or Sb in isolation. Sr atoms modify Mg_2_Si by preferentially adsorbing on {100} facets of Mg_2_Si crystals [19], and Sb atoms are attributed to the heterogeneous nucleation of primary Mg_2_Si crystals on Mg_3_Sb_2_, with the changing growth manners caused by the incorporation of Sb in the Mg_2_Si crystals, and preferential adsorption of Sb on the Mg_2_Si {100} surfaces to suppress the growth rate along the <100> directions [20].

**Figure 4:**
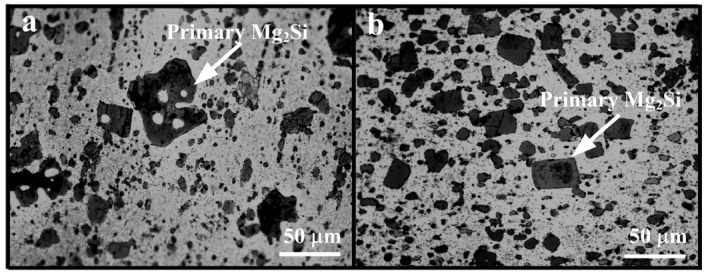
Microstructures of primary Mg_2_Si in hot-extruded (extruding temperature: 460 °C) and T6 treated (solution temperature and time: 500 °C/2h, aging temperature and time: 175 °C/8h) alloys: (**a**) Al–18Mg_2_Si–4.5Cu, (**b**) Al–18Mg_2_Si–4.5Cu–0.2(Sr–Sb).

The OM microstructure of hot-extruded Al–18Mg_2_Si–4.5Cu alloys before and after adding 0.2 wt % Sr–Sb are shown in Figure 4a,b, respectively. After hot extrusion and heat treatment, the edge of primary Mg_2_Si become more smooth and the particle distribution of primary Mg_2_Si was more uniform (Figure 4a,b) than that in as-cast alloys (Figure 2a,b). Moreover, the eutectic Mg_2_Si turns to granular shapes in Al–18Mg_2_Si–4.5Cu, as shown in Figure 4. The rounded edge could reduce the possibility of stress concentration between the reinforced Mg_2_Si particles and the matrix materials, which was beneficial in improving the mechanical properties of alloys.

**Figure 5:**
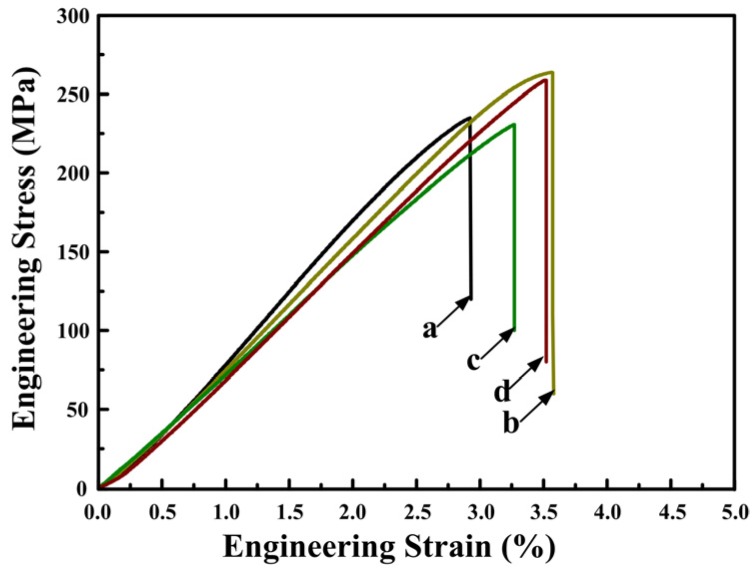
Engineering stress–stain curves of hot-extruded and T6 treated alloys: (**a**) Al–18Mg_2_Si–4.5Cu tensile at room temperature; (**b**) Al–18Mg_2_Si–4.5Cu–0.2(Sr–Sb) tensile at room temperature; (**c**) Al–18Mg_2_Si–4.5Cu tensile at 100 °C; (**d**) Al–18Mg_2_Si–4.5Cu–0.2(Sr–Sb) tensile at 100 °C.

To elucidate the effect of the Sr–Sb complex modifying on the mechanical properties, tensile tests were performed for the hot-extruded alloys. Figure 5a,d presents the engineering stress–engineering strain curves of Al–18Mg_2_Si–4.5Cu alloys with and without 0.2 wt % Sr–Sb at room temperature and at 100 °C. Table 1 shows the average ultimate tensile strengths, fracture elongations and the microhardness of the experimental alloys at room temperature and 100 °C. The addition of 0.2 wt % Sr–Sb increased the UTS and elongation-to-fracture of the Al–18Mg_2_Si–4.5Cu alloys both at room temperature and 100 °C. Under room temperature, the UTS increases from 229 to 288 MPa in Al–18Mg_2_Si–4.5Cu alloys after modification, and the elongation to fracture only slightly improves from 2.9% to 3.8%. As a comparison, Yeganeh *et al.* [8] and Wang *et al*. [21] have improved the ultimate tensile strength to 210 MPa by adding 1 wt % P in Al–25Mg_2_Si alloys, and to 283 MPa by adding 0.5 wt % Sb in Al–20Mg_2_Si alloys, respectively. Our experiment proves that the combined addition of only minor (0.2 wt %) Sr–Sb can get the better modification effect. Moreover, the microhardness of the matrix in our alloys is 16 Hv higher than that in Al–1.17Mg–0.57Si–0.5Cu (at %) alloys [22]. 

Then the samples were soaked at 100 °C for 10 min before tensile tests. With the addition of 0.2 wt % Sr–Sb, the UTS of Al–18Mg_2_Si–4.5Cu alloys increases from 231 to 272 MPa, and fracture elongation increases from 3.3% to 3.7%. Generally, the mechanical property of conventional aluminum alloy drill pipe decreased rapidly when soaking at 100 °C under ultra-deep drilling wells [23]. The Sr–Sb addition significantly improved the ultimate tensile properties of the composites by 17% even at 100 °C, which will promote their high temperature applications, e.g., serving on the bottom of drilling wells for gas and oil. Therefore, the thermally stable Mg_2_Si phase can effectively improve the high temperature mechanical properties of aluminum alloys [3], which makes it possible to manufacture drilling pipes for ultra-deep exploration with Mg_2_Si/Al alloys.

The increase of tensile strength after modification of Sr–Sb is mainly attributed to the change of primary Mg_2_Si morphologies and the decrease of their grain sizes. According to Chen *et al.* [24], adding 4.5 wt % Cu is mainly to increase the mechanical properties of the alloys. It is also confirmed by Zoqui *et al.* [25] that the Cu–rich intermetallics, *i.e.*, Al_2_Cu phase (See Figure 1), contributes to improving the tensile strength of the alloys.

In the hot-extruded and T6 treated Al–18Mg_2_Si–4.5Cu alloys, the microhardness of α-Al matrix improves slightly from 169 to 173 Hv after the modification of 0.2 wt % Sr–Sb. This improvement in the hot-extruded and T6 treated alloys is much less compared with the as-cast alloys, which increases from 116 to 141 Hv. The reason for this is that the hard dendritic Mg_2_Si particles are broken up by the extrusion process, contributing to more refinement and uniform distribution of Mg_2_Si phases in the matrix.

**Figure 6:**
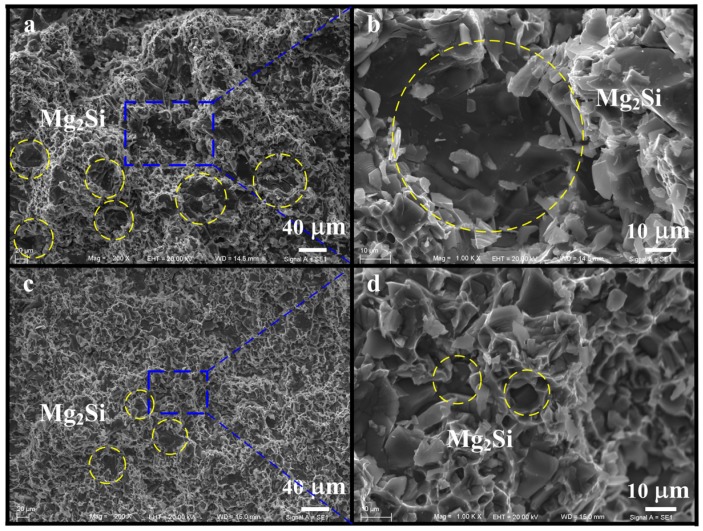
The SEM fracture morphology of hot-extruded and T6 treated alloys tensile at room temperature: (**a**,**b**) Al–18Mg_2_Si–4.5Cu; (**c**,**d**) Al–18Mg_2_Si–4.5Cu–0.2(Sr–Sb). (In yellow circles are primary Mg_2_Si particles).

For particulate reinforced alloys, their mechanical properties are significantly influenced by both reinforcement and matrix [15]. Typical SEM images of fracture surface morphologies tensiling at room temperature are shown in Figure 6a–d (with and without 0.2 wt % Sr–Sb). The modified alloy has more fine Mg_2_Si particles distributed in the α-Al matrix. A careful examination of this micrograph reveals a transition from brittle fracture to ductile fracture mode. The alloys before modification exhibit particles cracking rather than particle/matrix decohesion (See Figure 6b). The local stress concentration is produced and develops on the sharp straight boundaries of primary Mg_2_Si particles, which makes the linking of cracks rather easy; this explains high fragility and may cause low tensile strength of non-modified alloys. By contrast, the fracture surface of modified alloys show more and finer dimples, particles cracking and particle/matrix decohesion occurring at the same time (See Figure 6c). The modified primary Mg_2_Si and eutectic Mg_2_Si particles change to fine cube and short rod, lowering the local stress concentration. One part of decohesion occurs between the cubic Mg_2_Si particles and the matrix instead of within Mg_2_Si when the tensile strength increases. That is to say, some of the rupture has a ductile nature, indicating that the cracks hardly propagated though these precipitates and exhibit intergranular fracture. The morphology of Mg_2_Si has a critical effect on the mechanical properties of the alloys. The Mg_2_Si particles become finer, and the mechanical properties of the alloys are improved. Therefore, the variation in the morphology and size of Mg_2_Si particles resulted in brittle–ductile transition of the alloys.

## 4. Conclusions

After the combined addition of Sr–Sb, the morphology of primary Mg_2_Si in as-cast Al–18Mg_2_Si–4.5Cu alloys transforms from equiaxed dendrite to cube, and the particle size decreases from ~50 to ~20 μm. Eutectic Mg_2_Si changes from Chinese script to short rod shape. In hot-extruded Al–18Mg_2_Si–4.5Cu alloys, the edge of primary Mg_2_Si becomes more rounded and the particle distribution of primary Mg_2_Si is more uniform.

Through a complex modification of 0.2 wt % Sr–Sb, the UTS increases from 229 to 288 MPa in hot-extruded Al–18Mg_2_Si–4.5Cu alloys at room temperature, while it increases from 231 to 272 MPa at 100 °C. Microhardness increases from 116 to 141 Hv in as-cast alloys and from 169 to 173 Hv in hot-extruded alloys. The mechanical properties are strongly dependent on the morphology and size of Mg_2_Si.

## Figures and Tables

**Table 1:** The mechanical properties of hot-extruded and T6 treated Al–18Mg_2_Si–4.5Cu alloys with and without 0.2 wt % Sr–Sb addition tested at room temperature and at 100 °C.

Test Temperature	Materials	$\sigma_{b}$/MPa	$\delta_{f}$/%	Hardness/Hv	
				As-Cast	Hot-Extruded
RT	Al–18Mg_2_Si–4.5Cu	$229_{-7}^{+6}$	$2.9_{-0.2}^{+0.2}$	$116_{-10}^{+12}$	$169_{-9}^{+11}$
	Al–18Mg_2_Si–4.5Cu–0.2(Sr–Sb)	$288_{-4}^{+3}$	$3.8_{-0.6}^{+0.5}$	$141_{-12}^{+13}$	$173_{-11}^{+13}$
100 °C	Al–18Mg_2_Si–4.5Cu	$231_{-2}^{+1}$	$3.3_{-0.1}^{+0.2}$	-	-
	Al–18Mg_2_Si–4.5Cu–0.2(Sr–Sb)	$272_{-4}^{+5}$	$3.7_{-0.1}^{+0.1}$	-	-
